# A Systematic Comparison Identifies an ATP-Based Viability Assay as Most Suitable Read-Out for Drug Screening in Glioma Stem-Like Cells

**DOI:** 10.1155/2016/5623235

**Published:** 2016-05-05

**Authors:** A. Kleijn, J. J. Kloezeman, R. K. Balvers, M. van der Kaaij, C. M. F. Dirven, S. Leenstra, M. L. M. Lamfers

**Affiliations:** ^1^Department of Neurosurgery, Brain Tumor Center, Erasmus MC, 3015 CE Rotterdam, Netherlands; ^2^Department of Neurosurgery, St. Elisabeth Hospital, 5022 GC Tilburg, Netherlands

## Abstract

Serum-free culture methods for patient-derived primary glioma cultures, selecting for glioma stem-like cells (GSCs), are becoming the gold standard in neurooncology research. These GSCs can be implemented in drug screens to detect patient-specific responses, potentially bridging the translational gap to personalized medicine. Since numerous compounds are available, a rapid and reliable readout for drug efficacies is required. This can be done using approaches that measure viability, confluency, cytotoxicity, or apoptosis. To determine which assay is best suitable for drug screening, 10 different assays were systematically tested on established glioma cell lines and validated on a panel of GSCs. General applicability was assessed using distinct treatment modalities, being temozolomide, radiation, rapamycin, and the oncolytic adenovirus Delta24-RGD. The apoptosis and cytotoxicity assays did not unequivocally detect responses and were excluded from further testing. The NADH- and ATP-based viability assays revealed comparable readout for all treatments; however, the latter had smaller standard deviations and direct readout. Importantly, drugs that interfere with cell metabolism require alternative techniques such as confluency monitoring to accurately measure treatment effects. Taken together, our data suggest that the combination of ATP luminescence assays with confluency monitoring provides the most specific and reproducible readout for drug screening on primary GSCs.

## 1. Introduction

Malignant gliomas, in particular grade IV glioblastoma multiforme (GBM), are associated with a dismal prognosis despite current therapy consisting of surgery, chemotherapy, and radiation [1]. Improved treatment options are therefore urgently needed. In the past years a vast amount of antineoplastic agents have been developed which may potentially benefit glioma patients. To date, none of these promising preclinical agents have demonstrated an additional value to the current treatment regimen for which multiple reasons may contribute. First of all, GBM has been proven to consist of substantial interindividual and intraindividual heterogeneity resulting in highly variable responses to treatment [2–6]. In addition, a subpopulation of tumor initiating cells may contribute to both this heterogeneity and therapeutic resistance [7, 8]. Specific defined culture protocols have been developed to select for and propagate such cells, termed glioma stem-like cells (GSCs), from freshly dissociated patient tumor material. GSCs were found to better recapitulate the genotype and gene expression patterns of human glioblastomas [9–12]. Biobanking initiatives based on this model now enable the possibility to perform paneled GSC-based drug screen experiments to identify agents eligible for further testing in specific tumor subgroups. Based on these insights and developments, GSCs have become the preferred model for* in vitro* investigations into new therapeutic entities for malignant glioma and may provide an ideal platform for development of patient-tailored medicine.

To assess the potential of new compounds in the GSC model, a reproducible and rapid read-out of the treatment effect on glioma cells is essential. Multiple options for quantification of antitumor effects are available including cell counts, proliferation, viability, clonal growth, cytotoxicity, and apoptosis, using either microscopy-, absorbance-, fluorescence-, or luminescence-based assays.

Cell death can be induced through several mechanisms of which apoptosis, necrosis, senescence, and autophagy are to date the best characterized [13, 14]. While apoptosis is associated with the activation of caspases and loss of membrane integrity, autophagy is characterized by vacuolization and LC3 lipidation [15]. In contrast, senescent cells remain viable but acquire morphological changes and cease to synthesize DNA [14]. Ideally, an assay used for drug screening should be able to capture all these forms of cell death in a reliable manner, as the type of cell death induced by a new compound is not always known beforehand.

We tested 10 different assays in a standardized cell line-based* in vitro* model (U373 and T98) for the first selection of suitable assays, which were subsequently validated on GSC cultures. Four therapeutics were selected based on their distinct mechanisms of action in antitumor activity: temozolomide (TMZ), radiation, rapamycin, and the oncolytic adenovirus Delta24-RGD. TMZ and radiation comprise the current standard treatment regimen for glioma patients [1]. Rapamycin is an mTOR inhibitor belonging to the class of kinase inhibitors. This agent has received widespread attention during the last decade and is currently being tested in clinical trials for various cancer types including glioma [16, 17]. Delta24-RGD is an oncolytic adenovirus, which is currently being tested in clinical phase I/II trials for glioblastoma [18]. The cell lines U373 and T98 were selected for the initial assay screens based on their reported differential response to each of the therapeutic agents [19–22]. In addition, the selected treatments have been reported to induce various types of cell death [23–26].

In the current study, we sought to identify a drug screening assay which detects treatment response independently of the type of induced cell death. Furthermore, generated results must be reproducible: both intra-assay, with low standard deviations, and interassay. Also, the assay must have an adequate degree of discriminating capacity to clearly differentiate responder from nonresponder cell cultures. Next to these intrinsic capacities, low costs and easy handling are also important advantages for medium- to high-throughput drug screening assays.

To make a selection of best-suited assays for rapid drug screening, a broad range of assays, detecting caspases, LDH release, morphology, viability, or confluency, were evaluated. Assays that met the above-described criteria on the established cell lines were further validated on patient-derived GSC cultures.

## 2. Materials and Methods

### 2.1. Cell Culture

The glioma cell lines U373 and T98 were obtained from ECACC (Salisbury, UK) and ATCC (Manassas, VA), respectively. Cells were cultured in DMEM (Invitrogen, Breda, Netherlands), 10% FCS (Invitrogen), and 1% Penicillin/Streptomycin (Invitrogen). Primary glioma stem-like cell (GSC) cultures were derived from patient material as described previously [10] and cultured in DMEM/F12 (Invitrogen), 1% Penicillin/Streptomycin, 2% B27 (Invitrogen), bFGF, EGF (both 20 ng/mL, Tebu-Bio, Heerhugowaard, Netherlands), and heparin (5 *μ*g/mL, Sigma-Aldrich, Zwijndrecht, Netherlands). The use of patient tumor material was acquired with informed consent from patients as approved by the Institutional Review Board of the Erasmus Medical Center Rotterdam. Growth factor reduced extracellular matrix coating (ECM, diluted 1 : 10, 10 *μ*L/well, BD Biosciences, Breda, Netherlands) was used to grow GSC cultures in monolayers. For the validation of the assays, GSCs were plated at a density of 1000 cells/well in 96-well plates, which were precoated with ECM and treated according to the below described methods. The MGMT promoter methylation status of the cell culture was determined as described previously [27, 28].

### 2.2. Treatments

Both temozolomide (TMZ) (kindly provided by Sun Pharmaceutical Industries Ltd., Mumbai, India) and rapamycin (Calbiochem, Darmstadt, Germany) were dissolved in DMSO (Sigma-Aldrich) to a stock solution of 0.1 M and 1 mM, respectively. Further dilutions, 10, 50, and 100 *μ*M for TMZ and 10, 50, and 100 nM for rapamycin, were prepared in medium and added to the cells. The DMSO concentration never exceeded 0.1%. To test the effect of radiation therapy, cells were irradiated with 3 or 6 Gy using a Cesium-137 source.

For viral treatment, the tumor selective oncolytic adenovirus Delta24-RGD was used. The construction and production of this virus have been described previously [29, 30]. Dilutions for multiplicity of infection (MOI) 3, 10, 30 and 100 of Delta24-RGD were made in culture medium. The GSC cultures were treated with the GFP-expressing variant Delta24-GFP-RGD [31]; this variant was used to check for infection efficiency.

### 2.3. Apoptosis Assays

For the Caspase-GLO 3/7 assay (Promega, Leiden, Netherlands), U373 and T98 cells were seeded at a density of 10.000 cells/well. After 16 hours of incubation the treatment effect was measured according to the manufacturer's instructions using the Infinite 200 reader (Tecan, Männedorf, Switzerland).

As a positive control 20 nM of staurosporine (Merck Millipore, Billerica, MA) was added to the cells. Results are expressed as percentage of nontreated cells and error bars indicate standard deviation.

### 2.4. Clonogenic Assay

For the clonogenic assay the protocol described by Franken et al. was used [32]. Briefly, 1000 U373 cells, 500 T98 cells, or 500, 1000, or 2000 GSC cells were seeded in T25 flasks in duplicate and treated with TMZ, rapamycin, or radiation after 24 hours. After 10 days, cells were fixed and stained using 6% glutaraldehyde (Sigma-Aldrich) and 0.5% crystal violet (Sigma-Aldrich). The colonies, defined to consist of at least 50 cells, were counted by two independent persons. Plating efficiency was determined as the ratio of the number of colonies to the number of cells seeded. The surviving fraction was calculated by dividing the number of colonies by the plating efficiency and multiplying this by the number of cells seeded.

### 2.5. Cell Viability and Cytotoxicity Assays

U373 cells and T98 cells were seeded at a density of 500 cells in 96-well plates and incubated for 24 hours. Each experiment was performed in triplicate. For the cell proliferation assays (WST-1 (Roche, Basel, Schweiz), CellTiter-GLO, CellTiter-Fluor (Promega), and Alamar Blue (Invitrogen)) as well as the cytotoxicity assays (CytoTox-GLO, CytoTox-One (Promega)) treatment effect was measured after 5 or 6 days of incubation. These time points were selected based on pilot time course experiments showing clear treatment effects at these points with control untreated cells still growing in the exponential growth phase. All assays were performed according to the manufacturers' instructions. Absorption, luciferase, or fluorescent signals were measured using the Infinite 200 reader (Tecan, Männedorf, Switzerland).

Results from the cytotoxicity assays are expressed as percentage of induced cytotoxicity compared to the nontreated control; error bars indicate standard deviation. Results from the viability assays are expressed as percentage of the nontreated control and error bars indicate standard deviation.

### 2.6. Cell Count Assays

Cell confluency was determined using the live imaging system IncuCyte (Essen BioScience, Ann Arbor, Michigan, USA). To this end, cells were seeded and treated as described above, placed in the IncuCyte system inside the incubator, and followed for the entire incubation period. Every 3 hours the system acquired an image and confluency was measured using the algorithm in the IncuCyte software (Essen BioScience). Results are expressed as % confluency.

The crystal violet assay was performed as described previously [33]. In brief, cells were seeded and treated as described above. After 5 days of incubation the cells were fixed using 6% glutaraldehyde (Sigma-Aldrich) and 0.5% crystal violet (Sigma-Aldrich). The staining solution was removed and the plates were washed and dried overnight. The crystal violet staining was dissolved in 10% acetic acid and absorption was measured at 595 nm.

### 2.7. Analysis

For all assays, the discriminative ability (DA) was determined by calculating the ratio between the treatment effect of the responder and the treatment effect of the nonresponder at the highest dose. Student's *t*-test was used for significance testing; *p* values below 0.05 were considered significant (GraphPad Software, La Jolla, CA, USA).

## 3. Results

### 3.1. Apoptosis Assays and Cytotoxicity Assays

A detailed overview of all tested assays can be found in Supplemental Table  1 of the Supplementary Material available online at http://dx.doi.org/10.1155/2016/5623235. First, the apoptosis and cytotoxicity assays were tested on U373 and T98 cells. When apoptosis is induced by a certain treatment, increased cleavage of caspase 3/7 can be detected by an increase in luminescent signals compared to nontreated controls (indicated by dotted line in Supplemental Figure  1). None of the tested treatment modalities (Supplemental Figures  1(A–D)) induced an increase in caspase cleavage in either cell line, with the exception of the positive control staurosporine (Supplemental Figure  1(E)), which increases measured levels of apoptosis to 150% in U373 and 180% in T98 cells (*p* < 0.05).

Of the two tested cytotoxicity assays, the CytoTox-One assay did not detect the induced cytotoxicity in the responder cells for any of the tested treatments. The CytoTox-GLO assay did measure release of the dead-cell protease in treated cells; in both irradiated and TMZ treated cells a dose-dependent increase was noted (Supplemental Figure  2(A), T98 6 Gy *p* < 0.0001, Supplemental Figure  2(B), U373 100 *μ*M TMZ, *p* < 0.0001). Interestingly, treatment with rapamycin resulted in lower levels of LDH and death-cell protease release in U373 and T98, resulting in apparently lower cytotoxicity levels in treated compared to nontreated cells (Supplemental Figure  2(C)).

Overall, CytoTox-GLO assay did measure cytotoxicity, although not consistently in a dose-dependent or predicted manner. Based on these results, both the apoptosis and cytotoxicity assays were not further validated in primary GSCs.

### 3.2. Clonogenic Assay

**Figure 1:**
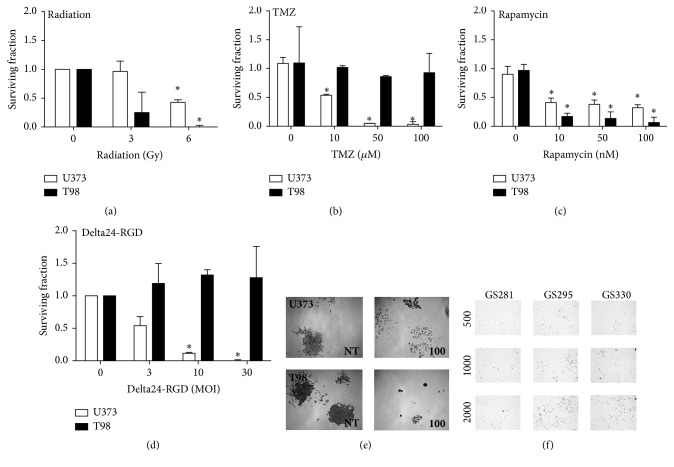
The clonogenic assay detects treatment effects in cell lines but not in GSCs. U373 cells (white bars) or T98 cells (black bars) were treated with radiation (a), TMZ (b), rapamycin (c), and Delta-24RGD (d) at different doses (*x*-axis). After 10 days clones were stained and counted by hand and the surviving fraction was calculated. Error bars indicate standard deviation, ^*∗*^ *p* < 0.05. Microscopic pictures of clones (e) treated with 100 nM rapamycin (right images) and nontreated clones (left images) of U373 cells (top row) and T98 cells (bottom row). Magnification ×10. Three primary GSCs (f) were tested for their ability to form clones over a 10-day period. Cells were plated at densities of 500, 1000, or 2000 cells/T25 flask. Magnification ×10.

The clonogenic assay has been described as the gold standard for radiation-induced cell death [32]. It measures the ability of single cells to proliferate and form clones after treatment. As expected, the effect of radiation on the cell lines U373 and T98 is clearly visible as a dose-dependent decrease in the surviving fraction (Figure 1(a) U373 6 Gy, *p* = 0.0031, T98 6 Gy, *p* < 0.0001) with a greater effect on the radiosensitive T98 cells. The differential sensitivity to TMZ of these cell lines was also detected. The surviving fraction of U373 declines to almost zero after treatment while T98 cells remain unaffected, in accordance with reported chemosensitivity of these cell lines [19] (Figure 1(b), *p* < 0.05). Rapamycin treatment lowered the surviving fraction in both cell lines (Figure 1(c), U373 100 nM, *p* = 0.003, T98 100 nM, *p* = 0.0048); however, rapamycin also induced morphological changes in the U373 clones. The presence of scattered small clusters or single cells, made the assessment of the clones difficult as can be seen in the microscopic pictures of Figure 1(e). Treatment with oncolytic virus Delta24-RGD resulted in complete loss of clone formation at the dose of MOI 10 in the permissive cell line U373 (Figure 1(d), *p* < 0.0001). This outcome most probably reflects ongoing viral replication during the incubation period of clone formation. T98 cells were, as expected, not affected by the viral treatment. Taken together, the clonogenic assay detects treatment-induced loss of proliferative capacity and allows discrimination between responders and nonresponders. However, when a drug induces morphological changes to the cells, results can be more difficult to interpret. To assess the potential of the clonogenic assay in GSCs, three GSC cultures were seeded at varying cell densities in T25 flasks and grown for 10 days. As shown in Figure 1(f), all three GSC lines grew in dispersed manner as single cells and no clones were formed in this period (Figure 1(f)). We therefore conclude that the clonogenic assay is not suitable as drug efficacy read-out for GSC cultures.

### 3.3. Viability Assays

**Figure 2 fig2:**
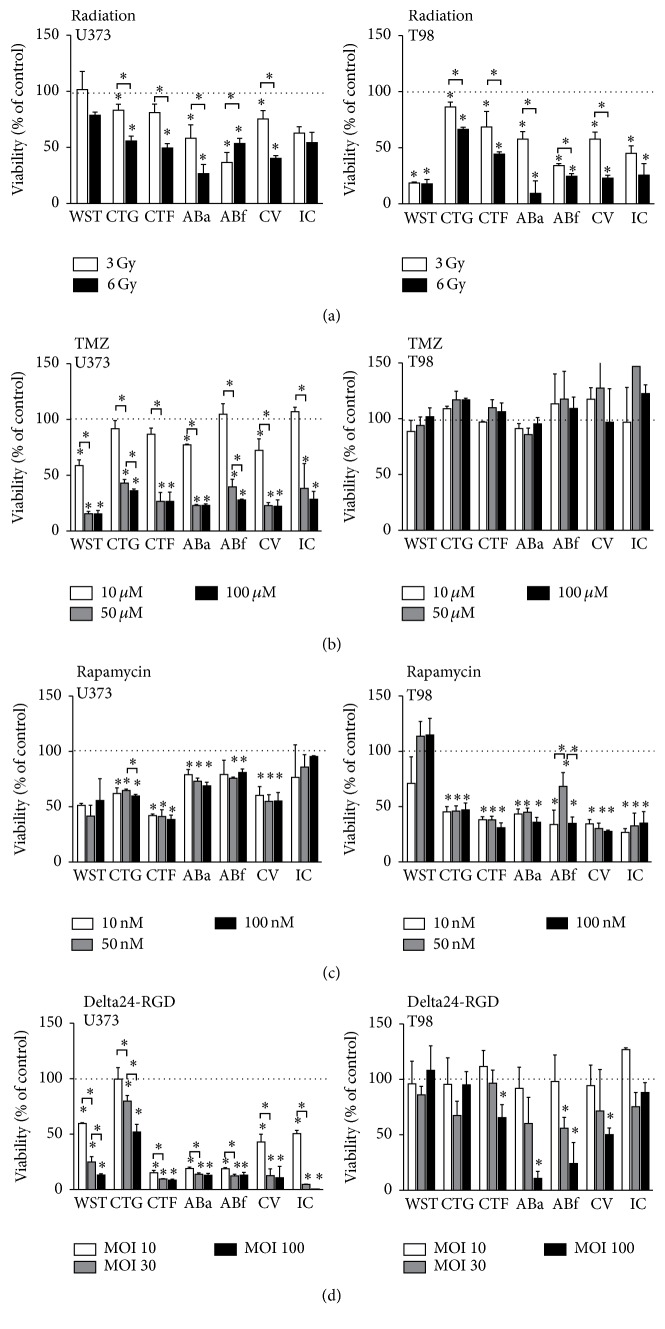
Comparison of all tested viability assays. U373 cells (left column) or T98 cells (right column) were treated with (a) radiation (3 Gy (white bars), 6 Gy (black bars)), (b) TMZ (10 *μ*M (white bars), 50 *μ*M (grey bars), and 100 *μ*M (black bars)), (c) rapamycin (10 nM (white bars), 50 nM (grey bars), and 100 nM (black bars)), or (d) Delta24-RGD (MOI 10 (white bars), MOI 30 (grey bars), and MOI 100 (black bars)). Read-out was after 5 or 6 days with the WST-1 assay (WST), CellTiter-GLO assay (CTG), CellTiter-Fluor assay (CTF), Alamar Blue assay with absorbance (ABa), Alamar Blue assay with fluorescence (ABf), crystal violet (CV) assay, or IncuCyte (IC). Results are expressed as mean percentage of viability compared to nontreated controls (100%, dotted line). Error bars indicate standard deviation, ^*∗*^ *p* < 0.05.

A panel of viability and proliferation assays was tested measuring either metabolic activity, live cell protease activity, actual cell count, or monolayer confluency (Figure 2). All assays demonstrated a decrease in viability after radiation in a dose-dependent manner; however, the degree of viability loss demonstrated large interassay variability. For example, viability of radiosensitive T98 cells after 3 Gy irradiation varied between 85% (CTG assay) and 20% (WST assay) (Figure 2(a), right graph). Overall, results of all assays reflect that T98 cells are more sensitive for radiation than U373 cells.

The differential effect of TMZ treatment on the responder U373 and nonresponder T98 cells was also detected by all tested assays (Figure 2(b)). The viability of U373 cells decreased in a dose-dependent manner between 10 *μ*M (white bars) and 50 *μ*M (grey bars). The largest variability between the different assay results is detected at 10 *μ*M TMZ, especially between the viability as measured by WST (40% decrease) and confluence as measured by the IncuCyte (IC; no decrease). None of the tested assays reported a decrease in viability of the resistant T98 cells upon TMZ treatment (Figure 2(b), right graph).

**Figure 3:**
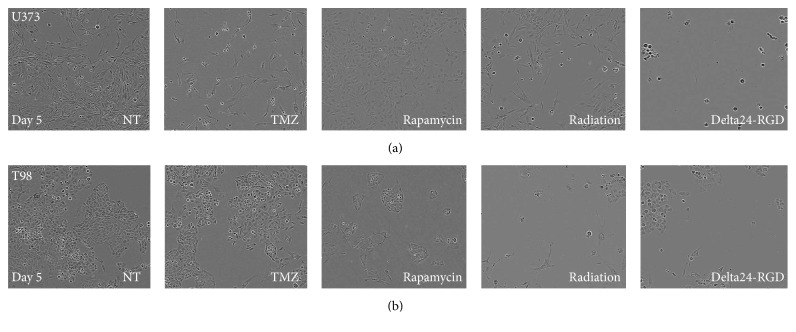
Treatment induces morphological changes in U373 and T98 cells. Microscopic pictures made by the IncuCyte of U373 (a) or T98 cells (b) showing nontreated cells (NT) or treated cells with TMZ, rapamycin, radiation, or Delta24-RGD after 5 days. The highest tested dose is shown, magnification ×10.

Analysis of the effects of rapamycin on U373 and T98 cells revealed larger interassay variability (Figure 2(c)). All assays revealed a reduction in viability in the rapamycin sensitive T98 cells, with the exception of the WST-1 assay. Also, the treatment effect of rapamycin on U373 was not detected consistently by all assays; viability loss ranged from approximately 60% (CellTiter-Fluor assay) to almost no reduction (IncuCyte). Microscopic analysis of the cells, using pictures generated by the IncuCyte, revealed morphological changes in the cells after rapamycin treatment. As shown in Figure 3, U373 cells treated with rapamycin were less spindle-shaped with increased cytoplasm and smaller cell protrusions. The T98 cells were reduced in number, grew in clusters, and were irregularly shaped.

The viability assays were also compared in their ability to detect the effects of oncolytic virus Delta24-RGD treatment, in particular because the virus induces a cytopathic effect leading to cell detachment (Figure 3, right panel), which may influence reproducible viability assessment. In general, all tested assays detected a dose-response effect in the sensitive U373 cells and much smaller treatment effects in the resistant T98 cells (Figure 2(d)). However, the ABa, the ABf, and the CV assays do detect reduced viability in the T98 cells treated with MOI 100. Of note, CTG levels were higher compared to the other three viability assays in U373. This probably results from the assay procedure that, contrary to the other substrate-based assays, does not require removal of supernatants in which detached but still metabolically active virus-infected cells reside.

Overall, viability and confluency assays are able to differentiate between treatment effects in a dose-dependent manner. However, treatments that affect the metabolism and/or morphology of cells or lead to detachment of cells may interfere with the results of viability assays.

### 3.4. Comparison of All Tested Assays

The results of all tested assays are summarized in Table 1. To quantify the discriminating ability (DA) of the assays, the ratios between the viability of the responder and the viability of the nonresponder were calculated for each treatment. For the assays that detect a viability reduction, values <1 indicate a predicted difference between responder and nonresponder: the closer to 0 the greater the discriminative power. For the cytotoxicity and apoptosis assays, which measure an induction compared to the controls, values >1 indicate a difference, the higher the better. The DA of the viability assays detecting the rapamycin effect ranged from 0.2 (clonogenic assay) to 2 (WST-1), while for TMZ it ranged between <0.1 (clonogenic assay) and 0.3 (CTG, CTF, and ABf), indicating a good discriminative ability of all tested assays for TMZ. The DA for the viability assays detecting radiation effects ranged from 0.2 (WST-1) to 1.2 (CTG). For Delta24-RGD it ranged from <0.1 (clonogenic assay, IncuCyte) to 0.5 (CTG, ABf).

Overall, results from the crystal violet assay, clonogenic assay, CellTiter-Fluor assay, Alamar Blue assay, and CellTiter-GLO assay were comparable for most treatments; however, the latter showed a better reproducibility with lower standard deviations and the advantage of a direct read-out after 10 minutes, important benefits in high-throughput drug screening. Also, detached cells residing in the supernatant are included in the analysis with CTG, which can be an advantage with certain treatments. The crystal violet assay has the extra advantage of cost-effectiveness.

### 3.5. Validation of Crystal Violet and CTG Assays on Primary Cultures

**Figure 4:**
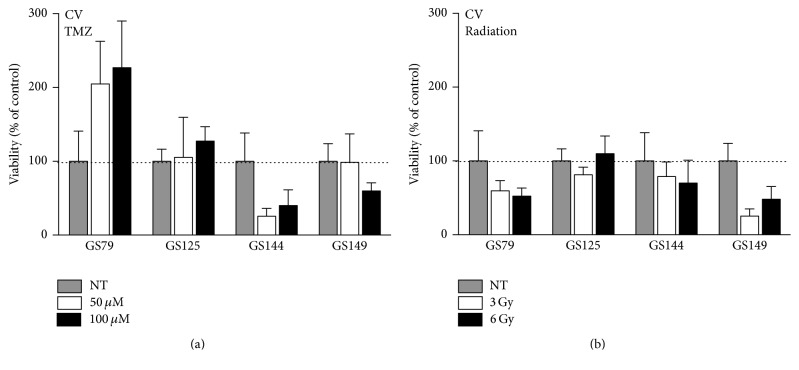
Validation of the crystal violet assay on primary glioma stem-like cell cultures. The crystal violet (CV) assay was applied on four primary glioma stem-like cell cultures. Cells were treated with 50 *μ*M (white bar) or 100 *μ*M (black bars) TMZ (a) and 3 Gy (white bars) or 6 Gy (black bars) radiation (b). Read-out was after 5 days. Error bars indicate standard deviation.

As mentioned before, the use of primary glioma stem-like cultures (GSC) holds potential benefits over established cell lines with regard to preclinical research on patient-specific therapies. Therefore, we selected the most reliable assays from the previous experiments, the CellTiter-GLO and the crystal violet assay, and validated these on a panel of GSCs (Supplemental Table  2) treated with TMZ, radiation, rapamycin, or Delta24-GFP-RGD. The crystal violet (CV) assay was first assessed for TMZ and radiation treatment on 4 GSC cultures and was found to be hampered by technical issues rendering it unreliable in this model (Figure 4). The crystal violet partly stains the extracellular matrix coating used for the GSC attachment, thereby leading to inaccurate staining and overestimation of cell numbers. Moreover, during the fixation and washing steps, cells detached and were lost, leading to large variation between triplets, as can be seen in the error bars of Figures 4(a) and 4(b). This drawback was most pronounced in the confluent nontreated wells, leading to an underestimation of treatment effects.

**Figure 5:**
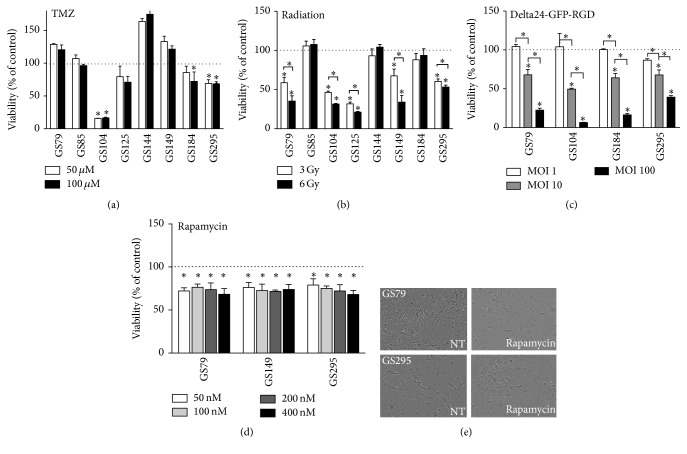
Validation of CellTiter-GLO assay on primary GSC cultures. The CellTiter-GLO assay was validated on primary glioma stem-like cell cultures. Cultures were treated with 50 *μ*M (white bar) or 100 *μ*M (black bars) TMZ (a), 3 Gy (white bars) or 6 Gy (black bars) radiation (b), MOI 1 (white bars), MOI 10 (grey bars), or MOI 100 (black bars) Delta-24-GFP-RGD (c), or 50, 100, 200, or 400 nM rapamycin (d). Read-out was after 5 days. Error bars indicate standard deviation, ^*∗*^ *p* < 0.05. Microscopic images of GS79 and GS295; nontreated (NT, left) and treated with 400 nM rapamycin (right) (e).

The CellTiter-GLO (CTG) assay was tested on 8 GSCs with three different treatment modalities (Figure 5). The 8 tested GSC lines (Supplemental Table  2) are all derived from primary tumors and are histologically diagnosed as glioblastoma (GBM) except for GS 144, which is derived from an anaplastic oligodendroglioma (OD, WHO grade III). A marker for TMZ response, the MGMT promoter methylation status, was determined in the GSCs and revealed both unmethylated and methylated cell cultures. The CTG assay measured treatment effects in a reliable manner in these lines, showing the differential response of primary cultures to the different treatment modalities. In general, the assay detected a larger decrease in viability in MGMT methylated GSCs compared to MGMT unmethylated GSCs after TMZ treatment (Figure 5(a), Supplemental Table  2). Dose-dependent responses were also detected after radiation and Delta24-GFP-RGD treatment (Figures 5(b) and 5(c)). In general, response of the virus-infected GSCs also correlated with level of infection as determined by GFP expression (not shown). For three GSC cultures, GS79, GS149, and GS295, the response to TMZ and rapamycin was compared between CTG and life cell confluence imaging. Rapamycin induced a small decrease in viability but not in a dose-dependent manner (Figure 5(d)). For both treatments, the IncuCyte confluence data correlated with the viability measurements by CTG (not shown). However, as observed in U373 and T98, the microscopic images revealed rapamycin-induced morphological changes in the GSCs. As shown in Figure 5(e), GS79 and GS295 cells became less spindle-shaped with smaller cell-protrusions and a more condensed phenotype.

Taken together, the CTG assay detected differential and dose-dependent response to all four treatment modalities in a panel of GSCs. However, for specific agents, microscopic imaging can help identify treatment effects not readily detected by ATP measurement.

## 4. Discussion

With the advent of personalized medicine and the increasing availability of new compounds, the development of an* in vitro* drug screening tool to select (patient-specific) effective drugs is increasingly relevant. The presented data provides insight into the nuances that exist between the existing viability, cytotoxicity, and apoptosis assays. A systematic comparison between these assays offers the opportunity to establish a protocol for practical, sensitive, and reproducible read-out of therapeutic efficacy of various types of treatment. We conclude that an ATP-based viability measurement in combination with microscopic imaging leads to robust detection of therapeutic efficacy for all four treatment modalities.

There are a large number of available cell death assays, which all focus on quite different biological phenomena, including viability, confluency, cytotoxicity, or apoptosis. However, defining cell death is a problem that is still under debate and there is no gold standard to assess cell death [34], complicating the comparison of the different assays. Nevertheless, the assays can be judged on several characteristics, independent of induced type of cell death, which defines their suitability for rapid drug screening. The most important requirement of the assay is that results should be reproducible and the assay should be sensitive with simple and fast read-out allowing rapid screening of large panels of compounds and cells.

In order to compare available assays in the absence of the expected heterogeneous responses of primary GSCs, a first selection was made using the established glioma cell lines U373 and T98, which both have well-defined and distinct response profiles to various therapies as reported in the literature. The cell line U373 is sensitive for TMZ and Delta24-RGD, whereas T98 is resistant [19, 22]. Conversely, T98 is more sensitive to radiation and rapamycin treatment compared to U373 [20, 21].

The tested apoptosis and cytotoxicity assays on these cell lines did not show reliable treatment responses (Supplemental Figures  1 and 2). Although apoptosis is described as a manner of cell death for the tested treatments, no induction was observed at the time point recommended by the manufacturer [23, 24]. Also the cytotoxicity assays failed to demonstrate increases in signals, possibly indicating high background levels of LDH release by these cells. Interestingly, rapamycin treatment actually led to lower levels of LDH release. This may result from a more general cell-cycle arrest state of the cells in response to rapamycin treatment [35, 36], as was also shown in the metabolic activity-based assays and microscopic images. As the induction of cell death pathways is both time- and treatment-dependent between (neoplastic) cells, pathway-dependent assays may be too heavily influenced by such variables for the acquisition of reproducible data. This suggests that these assays are not feasible for implementation in large-scale compound screens on panels of cell cultures but are more suitable to elucidate mechanisms of cell death in specific research questions.

All tested viability assays detected the differential treatment responses (Figure 4). The largest variability in results was observed after rapamycin treatment. As Figures 1(e) [Fig fig2] and 3 show, rapamycin induces morphological changes in the U373 and T98 cells whereas life cell confluency measurement detected only minimal treatment effects. Most viability assays, with the exception of the WST-1 assay, detected a large treatment effect. The described effects of rapamycin on cell metabolism could be responsible for this discrepancy, since the mTOR pathway is closely related to mitochondrial activity [37–39]. This indicates that combining a viability assay with microscopic imaging or confluence measurement is important to discriminate between treatment effects causing increased cell death and reduction in tumor cells versus treatment effects causing a reduction in metabolism.

Based on its reproducibility, sensitivity, and ease of use, the ATP-based CellTiter-GLO assay was further validated on a panel of GSCs. Differential and dose-dependent responses to TMZ, radiation, and oncolytic virus were observed. For TMZ, response of the GSCs was in accordance to that predicted by the MGMT status. As a confluency read-out to discriminate between metabolic and cytotoxic treatment effects, the crystal violet assay was applied. However, as the crystal violet also stains the extracellular matrix coating, necessary to grow GSCs in a monolayer, this assay was found to be unsuitable for use in GSC drug screening. Therefore, for this type of culture, live cell imaging is preferable, allowing detection of drug-induced morphology changes, as was observed for rapamycin and the virus-induced cytopathic effects leading to the presence of floating cells (Figures 3 and 5(e)). Also, live cell imaging generates confluency curves over time, which can help identify optimal time points for viability read-out. As GSC cultures demonstrate heterogeneous growth rates [40] and overconfluency perturbs reliable efficacy measurements, this is an important advantage of combining viability assays with imaging analysis in drug screening efforts on GSCs.

In conclusion, the present study has identified an ATP-based luminescent viability assay combined with microscopic imaging as the most reliable screening tool to detect the therapeutic effect in glioma cell lines and GSCs. This protocol can be utilized for high-throughput experiments in search of GSC response profiles for chemotherapeutics, small molecule inhibitors, oncolytic virotherapy, and radiation therapy. Although* in vitro* culture models have several shortcomings, for example, the delivery and toxicity of drugs being not taken into account, the results can provide important information on the response of the individual tumor cells to specific agents. By screening large panels of patient-derived cell cultures, identification of molecular features related to drug response may generate predictive profiles to a specific treatment [28, 41]. Validation of these profiles, as well as further issues such as delivery and toxicity, should be evaluated in* in vivo* models.

## Supplementary Material

Supplemental Table 1. Overview assays.
Supplemental Figure 1. The caspase 3/7 assay does not detect treatment effect in U373 and T98.
Supplemental Figure 2. Comparison of two cytotoxicity assays.
Supplemental Table 2. Characteristics of the used primary glioma stemElike cell cultures.

## Figures and Tables

**Table 1:** Summary of assay results.

Assay	Day	Radiation			TMZ			Rapamycin			Delta24-RGD		
		U373	T98	DA	U373	T98	DA	U373	T98	DA	U373	T98	DA
Caspase 3/7	16 hr	0	0	0.9	0	0	1	0	0	1	0	0	0.9
CytoONE	5/6	0	+	1.1	0	0	0.6	0	0	0.4	ND	ND	ND
CytoGLO	5	+++++	+++++	2.7	+	0	44.7	0	0	0.5	++++	0	2
Clonogenic	10	++++	+++++	0.3	+++++	0	<0.1	++++	+++++	0.2	+++++	0	<0.1
WST-1	5/6	++	++++	0.2	++++	0	0.2	+++	0	2	+++++	0	0.1
CTG	5/6	++	++	1.2	+++	0	0.3	++	+++	0.8	+++	0	0.5
CTF	5/6	+	++	0.9	++++	0	0.3	+++	+++	0.8	+++++	++	0.1
ABa	5	++++	+++++	0.4	++++	0	0.2	++	+++	0.5	+++++	+++++	1.2
ABf	5	+++	++++	0.5	++++	0	0.3	+	+++	0.4	+++++	++++	0.5
CV	5	+++	+++++	0.6	+++++	0	0.2	+++	++++	0.5	++++	+++	0.2
IncuCyte	5	+++++	+++	0.5	+++++	++	0.2	++	++	0.4	+++++	++	<0.1

Results are expressed by 0 = no effect, + = ↑↓ 10–20%, ++ = ↑↓ 20–40%, +++ = ↑↓ 40–60%, ++++ = ↑↓ 60–80%, and +++++ = ↑↓ 80–100%. The discriminative ability (DA) was calculated by dividing the viability at the highest dose of the responder cell line by the viability of the nonresponder cell line. CytoONE = CytoTox ONE®, CytoGLO = CytoTox-GLO®, CTG = CellTiter-GLO®, CTF = CellTiter-FLUOR®, ABa = Alamar Blue®, absorbance, ABf = Alamar Blue®, fluorescent, CV = crystal violet.

## References

[1] Stupp R., Mason W. P., Van Den Bent M. J. (2005). Radiotherapy plus concomitant and adjuvant temozolomide for glioblastoma. *New England Journal of Medicine*.

[2] Verhaak R. G. W., Hoadley K. A., Purdom E. (2010). ntegrated genomic analysis identifies clinically relevant subtypes of glioblastoma characterized by abnormalities in PDGFRA, IDH1, EGFR, and NF1. *Cancer Cell*.

[3] Liang Y., Diehn M., Watson N. (2005). Gene expression profiling reveals molecularly and clinically distinct subtypes of glioblastoma multiforme. *Proceedings of the National Academy of Sciences of the United States of America*.

[4] Piccirillo S. G. M., Combi R., Cajola L. (2009). Distinct pools of cancer stem-like cells coexist within human glioblastomas and display different tumorigenicity and independent genomic evolution. *Oncogene*.

[5] Sottoriva A., Spiteri I., Piccirillo S. G. M. (2013). Intratumor heterogeneity in human glioblastoma reflects cancer evolutionary dynamics. *Proceedings of the National Academy of Sciences of the United States of America*.

[6] Patel A. P., Tirosh I., Trombetta J. J. (2014). Single-cell RNA-seq highlights intratumoral heterogeneity in primary glioblastoma. *Science*.

[7] Hardee M. E., Marciscano A. E., Medina-Ramirez C. M. (2012). Resistance of glioblastoma-initiating cells to radiation mediated by the tumor microenvironment can be abolished by inhibiting transforming growth factor-*β*. *Cancer Research*.

[8] Blough M. D., Beauchamp D. C., Westgate M. R., Kelly J. J., Cairncross J. G. (2011). Effect of aberrant p53 function on temozolomide sensitivity of glioma cell lines and brain tumor initiating cells from glioblastoma. *Journal of Neuro-Oncology*.

[9] Lee J., Kotliarova S., Kotliarov Y. (2006). Tumor stem cells derived from glioblastomas cultured in bFGF and EGF more closely mirror the phenotype and genotype of primary tumors than do serum-cultured cell lines. *Cancer Cell*.

[10] Balvers R. K., Kleijn A., Kloezeman J. J. (2013). Serum-free culture success of glial tumors is related to specific molecular profiles and expression of extracellular matrix-associated gene modules. *Neuro-Oncology*.

[11] Galli R., Binda E., Orfanelli U. (2004). Isolation and characterization of tumorigenic, stem-like neural precursors from human glioblastoma. *Cancer Research*.

[12] Ernst A., Hofmann S., Ahmadi R. (2009). Genomic and expression profiling of glioblastoma stem cell-like spheroid cultures identifies novel tumor-relevant genes associated with survival. *Clinical Cancer Research*.

[13] Hotchkiss R. S., Strasser A., McDunn J. E., Swanson P. E. (2009). Mechanisms of disease: cell death. *New England Journal of Medicine*.

[14] Schmitt C. A. (2007). Cellular senescence and cancer treatment. *Biochimica et Biophysica Acta—Reviews on Cancer*.

[15] Galluzzi L., Maiuri M. C., Vitale I. (2007). Cell death modalities: classification and pathophysiological implications. *Cell Death and Differentiation*.

[16] Wen P. Y., Chang S. M., Lamborn K. R. (2014). Phase I/II study of erlotinib and temsirolimus for patients with recurrent malignant gliomas: North American Brain Tumor Consortium trial 04-02. *Neuro-Oncology*.

[17] Chinnaiyan P., Won M., Wen P. Y. (2013). RTOG 0913: a phase 1 study of daily everolimus (RAD001) in combination with radiation therapy and temozolomide in patients with newly diagnosed glioblastoma. *International Journal of Radiation Oncology Biology Physics*.

[18] http://clinicaltrials.gov/ct2/show/NCT01582516?term=delta24rgd+glioma&rank=2.

[19] Barazzuol L., Jena R., Burnet N. G. (2012). In vitro evaluation of combined temozolomide and radiotherapy using X rays and high-linear energy transfer radiation for glioblastoma. *Radiation Research*.

[20] Schoenherr D., Krueger S. A., Martin L., Marignol L., Wilson G. D., Marples B. (2013). Determining if low dose hyper-radiosensitivity (HRS) can be exploited to provide a therapeutic advantage: a cell line study in four glioblastoma multiforme (GBM) cell lines. *International Journal of Radiation Biology*.

[21] Takeuchi H., Kondo Y., Fujiwara K. (2005). Synergistic augmentation of rapamycin-induced autophagy in malignant glioma cells by phosphatidylinositol 3-kinase/protein kinase B inhibitors. *Cancer Research*.

[22] Ito H., Aoki H., Kuhnel F. (2006). Autophagic cell death of malignant glioma cells induced by a conditionally replicating adenovirus. *Journal of the National Cancer Institute*.

[23] Knizhnik A. V., Roos W. P., Nikolova T. (2013). Survival and death strategies in glioma cells: autophagy, senescence and apoptosis triggered by a single type of temozolomide-induced DNA damage. *PLoS ONE*.

[24] Jiang H., White E. J., Ríos-Vicil C. I., Xu J., Gomez-Manzano C., Fueyo J. (2011). Human adenovirus type 5 induces cell lysis through autophagy and autophagy-triggered caspase activity. *Journal of Virology*.

[25] Castedo M., Ferri K. F., Kroemer G. (2002). Mammalian target of rapamycin (mTOR): Pro- and anti-apoptotic. *Cell Death and Differentiation*.

[26] Cohen-Jonathan E., Bernhard E. J., McKenna W. G. (1999). How does radiation kill cells?. *Current Opinion in Chemical Biology*.

[27] Balvers R. K., Lamfers M. L. M., Kloezeman J. J. (2015). ABT-888 enhances cytotoxic effects of temozolomide independent of MGMT status in serum free cultured glioma cells. *Journal of Translational Medicine*.

[28] Berghauser Pont L. M., Spoor J. K., Venkatesan S. (2014). The Bcl-2 inhibitor Obatoclax overcomes resistance to histone deacetylase inhibitors SAHA and LBH589 as radiosensitizers in patient-derived glioblastoma stem-like cells. *Genes & Cancer*.

[29] Suzuki K., Fueyo J., Krasnykh V., Reynolds P. N., Curiel D. T., Alemany R. (2001). A conditionally replicative adenovirus with enhanced infectivity shows improved oncolytic potency. *Clinical Cancer Research*.

[30] Lamfers M. L. M., Grill J., Dirven C. M. F. (2002). Potential of the conditionally replicative adenovirus Ad5-Delta24RGD in the treatment of malignant gliomas and its enhanced effect with radiotherapy. *Cancer Research*.

[31] Berghauser Pont L. M., Kleijn A., Kloezeman J. J. (2015). The HDAC inhibitors scriptaid and LBH589 combined with the oncolytic virus Delta24-RGD exert enhanced anti-tumor efficacy in patient-derived glioblastoma cells. *PLoS ONE*.

[32] Franken N. A. P., Rodermond H. M., Stap J., Haveman J., van Bree C. (2006). Clonogenic assay of cells in vitro. *Nature Protocols*.

[33] Idema S., Geldof A. A., Dirven C. M. F. (2007). Evaluation of adenoviral oncolytic effect on glioma spheroids by 18F-DG positron-emission tomography. *Oncology Research*.

[34] Galluzzi L., Bravo-San Pedro J. M., Vitale I. (2015). Essential versus accessory aspects of cell death: recommendations of the NCCD 2015. *Cell Death and Differentiation*.

[35] Fingar D. C., Richardson C. J., Tee A. R., Cheatham L., Tsou C., Blenis J. (2004). mTOR controls cell cycle progression through its cell growth effectors S6K1 and 4E-BP1/eukaryotic translation initiation factor 4E. *Molecular and Cellular Biology*.

[36] Li J., Kim S. G., Blenis J. (2014). Rapamycin: one drug, many effects. *Cell Metabolism*.

[37] Yecies J. L., Manning B. D. (2011). Transcriptional control of cellular metabolism by mtor signaling. *Cancer Research*.

[38] Stepanenko A. A., Dmitrenko V. V. (2015). Pitfalls of the MTT assay: direct and off-target effects of inhibitors can result in over/underestimation of cell viability. *Gene*.

[39] Ramanathan A., Schreiber S. L. (2009). Direct control of mitochondrial function by mTOR. *Proceedings of the National Academy of Sciences of the United States of America*.

[40] Pollard S. M., Yoshikawa K., Clarke I. D. (2009). Glioma stem cell lines expanded in adherent culture have tumor-specific phenotypes and are suitable for chemical and genetic screens. *Cell Stem Cell*.

[41] Berghauser Pont L. M., Balvers R. K., Kloezeman J. J. (2015). *In vitro* screening of clinical drugs identifies sensitizers of oncolytic viral therapy in glioblastoma stem-like cells. *Gene Therapy*.

