# Supplementary material for: A Systematic Comparison Identifies an ATP-Based Viability Assay as Most Suitable Read-Out for Drug Screening in Glioma Stem-Like Cells
Source: Stem Cells Int. 2016 May 5;2016:5623235. doi: 10.1155/2016/5623235 (PMC4871979; doi:10.1155/2016/5623235)
Supplement: Supplementary file 1 — Supplemental Table 1. Overview assays. Supplemental Figure 1. The caspase 3/7 assay does not detect treatment effect in U373 and T98. Supplemental Figure 2. Comparison of two cytotoxicity assays. Supplemental Table 2. Characteristics of the used primary glioma stemElike cell cultures. [file 5623235.f1.zip › Description.docx]

**Supplementary Material**

*Supplemental Table 1 Overview assays*

This table provides an overview of the characteristics of assays applied in this study. Five

different viability assays, two cell proliferation assays, one apoptosis assay and two cytotoxicity assays, based on either microscopy, absorbance, fluorescence or luminescence, were tested. The incubation period of the assays varies from 10 minutes to 4 hours.

*Supplemental Figure 1 The caspase 3/7 assay does not detect treatment effect in U373 and T98*

Induction of apoptosis leads to cleavage of caspase 3/7 which can be measured by an increase in luminescent signal compared to nonDtreated controls (indicated by the dotted line). U373 and T98 cells were treated with different doses of radiation (figure1A), TMZ (figure1B), rapamycin (figure1C), Delta24-RGD (figure 1D) and staurosporine (as a positive control, figure 1E) and assay read-out was performed at 16 hours post-treatment as indicated by manufacturer’s instructions. No increase in apoptosis was detected in either cell line in any of the tested treatments, with the exception of the positive control staurosporine which increased levels of apoptosis to 150% in U373 and 180% in T98 cells (p < 0.05).

*Supplemental Figure 2 Comparison of two cytotoxicity assays*

Two cytotoxicity assays were tested; the CytoTox-ONE assay (CytoOne) measures LDH release in a fluorescent manner and the CytoTox-GLO assay (CytoGLO) measures activity of an unspecified dead-cell protease by luminescence. Both markers are released from cells after loss of membrane integrity. The effects of radiation, TMZ and rapamycin on T98 and U373 cells were assessed using both cytotoxicity assays (figure 2A-C). Whereas the CytoOne assay did not detect treatment effects for any of the therapeutic modalities (figure 2A-C, all p-values p>0.05), the CytoGLO assay did measure release of the dead-cell protease in treated cells. In irradiated cells a dose dependent increase was noted, particularly in the radiosensitive T98 cells (figure 2A, T98 6 Gy p < 0.0001). Also, after TMZ treatment, the CytoGLO showed induction of cytotoxicity in the TMZDsensitive U373 cells but not in the resistant T98 cells (figure 2B, U373

100 μM TMZ p<0.0001), whereas CytoOne did not detect TMZ-induced cytotoxicity in either cell line. Treatment with the oncolytic adenovirus Delta24-RGD was only tested with CytoTox-GLO which revealed a doseDdependent increase in cell death signal in de more susceptible U373 cells (figure 2D, not significant) whereas signals maintained at control levels in the resistant T98 cells.

Overall, the CytoTox-One assay did not detect the induced cytotoxicity in responder cells for any of the tested treatments, while the CytoTox-GLO assay did measure cytotoxicity, although not (consistently) in a dose-dependent or predicted manner.

*Supplemental Table 2 Characteristics of the used primary glioma stemElike cell cultures*

Overview of the primary glioma stem-like cell cultures applied in the current study, showing whether they are derived from primary or recurrent tumors, their histological diagnosis (GBM = glioblastoma multiforme, OD = oligodendroglioma) and the methylation status of the MGMT promoter (methylated or unmethylated) in the cell culture.
