# Supplementary material for: A Systematic Comparison Identifies an ATP-Based Viability Assay as Most Suitable Read-Out for Drug Screening in Glioma Stem-Like Cells
Source: Stem Cells Int. 2016 May 5;2016:5623235. doi: 10.1155/2016/5623235 (PMC4871979; doi:10.1155/2016/5623235)
Supplement: Supplementary file 1 — Supplemental Table 1. Overview assays. Supplemental Figure 1. The caspase 3/7 assay does not detect treatment effect in U373 and T98. Supplemental Figure 2. Comparison of two cytotoxicity assays. Supplemental Table 2. Characteristics of the used primary glioma stemElike cell cultures. [file 5623235.f1.zip › suppl material.pdf]

Supplemental Table 1: Assay characteristics

Name	Type	Based on	Half life*	Signal type	Incubation period	Result
Alamar Blue	Cell viability	Conversion of resazurin to resorufin by viable cells	no	C/F	1-4 hours	Viability
CellTiter-Glo	Cell viability	ATP based luciferase reaction	no	L	10 minutes	Viability
CellTiter-Fluor	Cell viability	Active live-cell protease based cleavage of fluorogenic substrate	no	F	30 minutes – 3 hours	Viability
Crystal Violet	Cell viability	Cell count	-	C	30 minutes	Viability
WST-1	Cell viability	Tetrazolium cleavage dependent on NAD(P)H production	no	C	1 -4 hours	Viability
Incucyte	Cell proliferation	Cell Confluency	-	-	-	% of confluency
Clonogenic Assay	Cell proliferation	Ability to form clones	-	-	30 minutes	Amount of clones<br>plating efficiency<br>surviving fraction
Caspase-Glo 3/7	Apoptosis	Caspase-3/7 activity	≥ 5 hr	L	30 min – 3 hours	Induced apoptosis
CytoTox-Glo	Cytotoxicity	Dead-cell protease based cleavage of luminogenic substrate	> 10 hr	L	30 minutes	Cytotoxicity<br>Viability
CytoTox-One	Cytotoxicity	LDH based conversion of resazurin to resorufin	9 hr	F	10 minutes	Cytotoxicity

Supplemental Figure 1

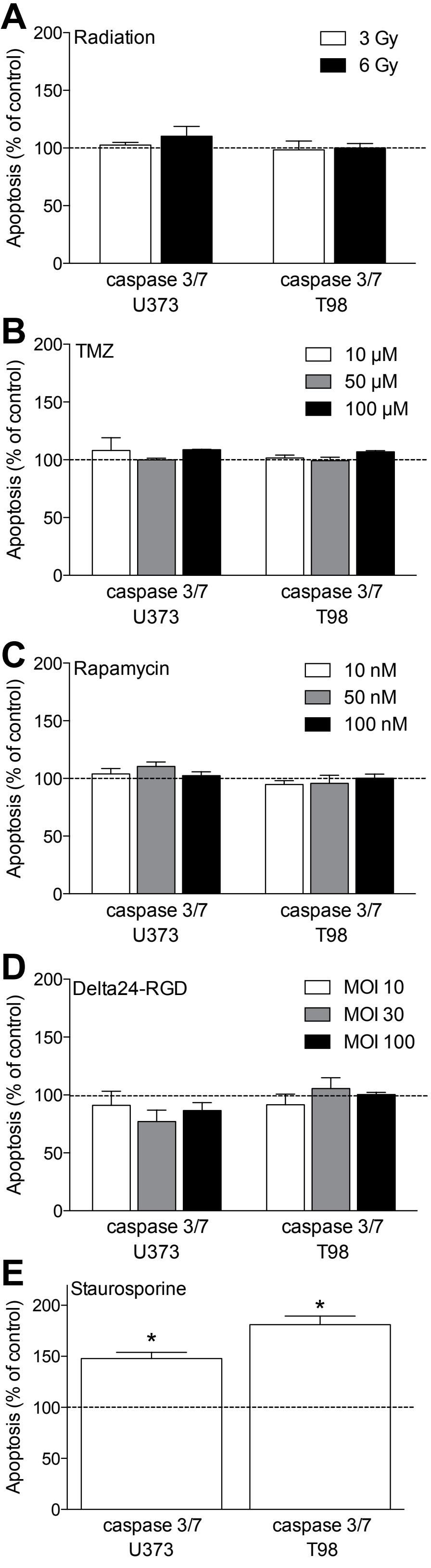

Supplemental Figure 2

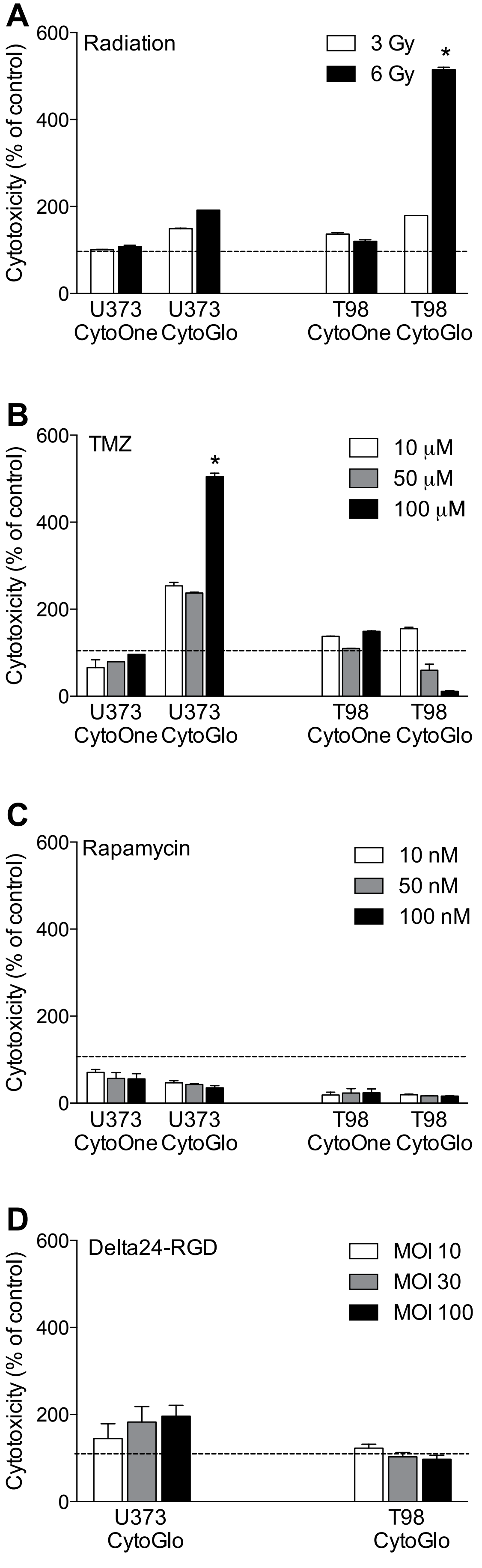

Supplemental Table 2

GS#	*Primary/Recurrent*	*Diagnosis*	*MGMT status*
GS79	Primary	GBM	Unmethylated
GS85	Primary	GBM	Methylated
GS104	Primary	GBM	Methylated
GS125	Primary	GBM	Unmethylated
GS144	Primary	Anaplastic OD	Not determined
GS149	Primary	GBM	Unmethylated
GS184	Primary	GBM	Methylated
GS281	Primary	GBM	Unmethylated
GS295	Primary	GBM	Methylated
GS330	Primary	GBM	Methylated
